# Associations between effort–reward imbalance and risk of burnout among Swedish physicians

**DOI:** 10.1093/occmed/kqae039

**Published:** 2024-07-06

**Authors:** F Christiansen, B E Gynning, A Lashari, G Johansson, E Brulin

**Affiliations:** Unit of Occupational Medicine, Institute of Environmental Medicine, Karolinska Institutet, 171 77 Stockholm, Sweden; Unit of Occupational Medicine, Institute of Environmental Medicine, Karolinska Institutet, 171 77 Stockholm, Sweden; Unit of Occupational Medicine, Institute of Environmental Medicine, Karolinska Institutet, 171 77 Stockholm, Sweden; Unit of Occupational Medicine, Institute of Environmental Medicine, Karolinska Institutet, 171 77 Stockholm, Sweden; Unit of Occupational Medicine, Institute of Environmental Medicine, Karolinska Institutet, 171 77 Stockholm, Sweden

## Abstract

**Background:**

The high prevalence of burnout among Swedish physicians may have several possible effects on individuals and society. However, further investigations of work-related factors associated with the risk of burnout among Swedish physicians are needed.

**Aims:**

We aimed to study the associations between psychosocial work factors, based on the effort–reward imbalance (ERI) model, and the risk of burnout among Swedish physicians.

**Methods:**

A representative sample of 7200 Swedish physicians was invited in 2021. Data were gathered through questionnaires, with a response rate of 41%. Logistic regression models were used to study the associations between exposure to ERI and the risk of burnout.

**Results:**

Approximately 62% of Swedish physicians were exposed to a high ERI. Exposure to a high ERI was associated with 11 times increased risk (95% confidence interval 6.5–20.0) of burnout in adjusted models. Large variations in the prevalence of ERI and risk of burnout across sociodemographic and occupational factors were identified, particularly across different clinical specialties.

**Conclusions:**

A majority of Swedish physicians were exposed to high levels of work-related stress, strongly associated with an increased risk of burnout. This population-based cross-sectional study underlines the need to further study variations of work-related stress across clinical specialties and to monitor occupational health among physicians longitudinally.

## Teaser

Work-related stress and burnout among physicians are common issues globally. This study investigated the associations between work stress, based on the effort–reward imbalance (ERI) model, and the risk of burnout among Swedish physicians. We found that a majority of Swedish physicians were exposed to high ERI. Exposure to ERI was strongly associated with an increased risk of burnout. Future studies should further explore work-related stress across clinical specialties and monitor occupational health among physicians longitudinally.

**Key learning points**

**What is already known about this subject:**

◦ The high prevalence of burnout among physicians globally may have several possible effects on individuals and society.
◦ Recently, a high prevalence of burnout among Swedish physicians was identified.
◦ Increased knowledge of work-related stress and contributing factors to burnout among Swedish physicians is warranted.

**What this study adds:**

◦ The present study adds to the knowledge of work-related stress among Swedish physicians across several occupational and sociodemographic factors.
◦ A majority of Swedish physicians were exposed to high levels of work-related stress (i.e. effort–reward imbalance) was strongly associated with an increased risk of burnout.
◦ There were large variations in experienced effort–reward imbalance and risk of burnout across different groups of Swedish physicians.

**What impact this may have on practice or policy:**

◦ Future studies should further investigate the observed variations in work-related stress across different clinical specialties and explore individual experiences of effort–reward imbalance among physicians.
◦ This study underlines the need to monitor occupational health among physicians longitudinally using consistent methods, both as local practice and for research purposes.

## Introduction

Burnout is a state characterized by a prolonged negative psychological response to various stressors at work [1]. According to international research, the prevalence of burnout among physicians is high [2]. A systematic review of 182 studies across 45 countries reported that an average of 67% of physicians show symptoms of burnout [3]. The high prevalence of burnout among physicians is alarming due to the possible detrimental effects on both individual and societal levels [2]. Recently, Hagqvist *et al*. identified a prevalence of (symptoms of) burnout in up to 28% of Swedish physicians, with significant variations across different specialties, work sites and technical ranks [4,5]. These findings suggest underlying work-related contributors to physician burnout that require further investigation.

There are several possible psychosocial work factors related to occupational burnout. In Swedish healthcare, organizational changes during the past decades have resulted in increased job strain among physicians. For example, increased work tasks and altered work characteristics have limited the ability to work efficiently [6]. In addition, the positive exchange for work efforts among physicians has decreased. Specifically, opportunities to engage in research and further education have been reduced [6], and low economic rewards, particularly for junior physicians, have been underlined in several opinion articles in the ongoing public debate [7,8]. These circumstances suggest an imbalance between occupational efforts and rewards among Swedish physicians, aligning with the effort–reward imbalance (ERI) model [9,10]. The ERI model stipulates a link between imbalances in experienced job efforts (e.g. the burden of work tasks, time pressure, responsibilities, etc.) versus rewards (e.g. income, career development opportunities, status influence, etc.) and various health outcomes, including burnout [10]. The ERI model has been widely used in previous research on occupational stress in healthcare settings, where high rates of ERI have been identified in several countries [11,12].

Previous Swedish studies have described poor working conditions for physicians [13,14]. However, the current knowledge of associations between work-related stress and health outcomes among Swedish physicians is limited [4,5]. Therefore, in this study, we aimed to investigate the associations between psychosocial work factors (i.e. ERI) and the risk of burnout among Swedish physicians.

## Methods

The present cross-sectional study was based on a representative sample of 7200 Swedish physicians in 2021, comprising physicians from various medical and surgical fields and different healthcare types (i.e. hospitals and primary care facilities). The sample was drawn from The Swedish Occupational Register, reflecting the entire occupational population as of 2018, provided by Statistics Sweden, the government agency responsible for official national data and statistics [15]. We employed a stratified random sampling method based on 12 strata, including geographics (six administrative healthcare regions) and work site (primary care facility or hospital). Inclusion criteria were actively working as a physician in Swedish healthcare at any point during the previous year. A total of 501 did not meet the inclusion criteria and were removed from the sample, leaving 6699 participants. Statistics Sweden conducted the data collection procedure between February and May 2021. In total, 2761 respondents submitted the questionnaire (response rate = 41%). For an extensive description of the data collection procedure, please see Hagqvist *et al*. [4]. The study was approved by the Swedish Ethical Review Authority (2020-06613).

**Figure 1.**
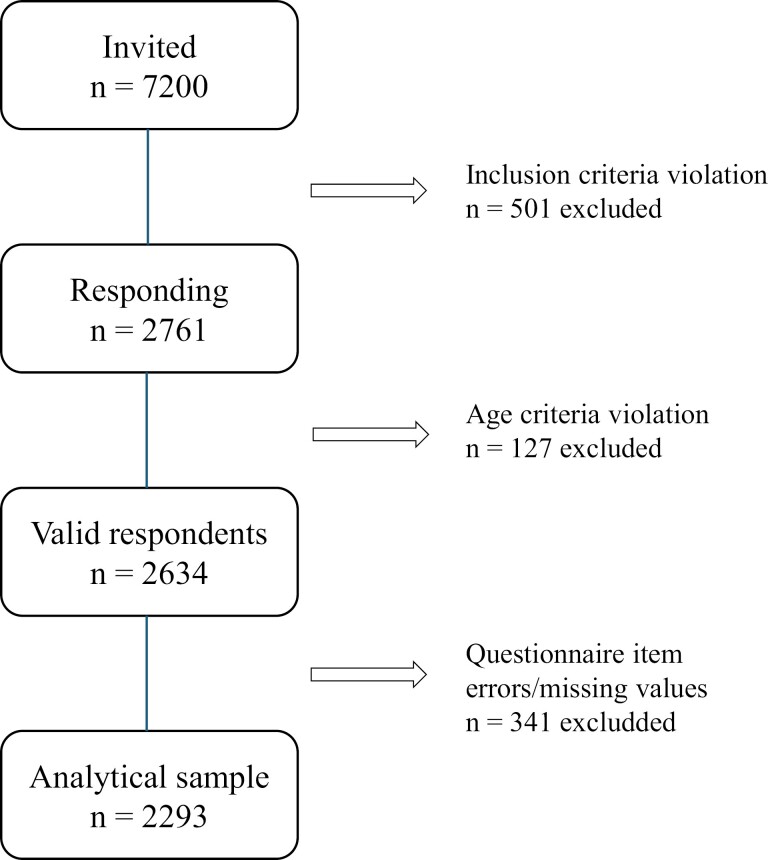
Flowchart of participants in the analytical sample of the present study.

In the present study, we restricted the sample to those aged <70, resulting in excluding 127 participants. An additional 210 participants were excluded due to questionnaire submission errors or missing data (figure 1). Of the eligible participants, 2293 had complete data for further analyses (mean age 46 years; females 55%).

Job efforts (*E*) and job rewards (*R*) were assessed using the validated ERI questionnaire [10], comprising separate scales with three items addressing *E* and seven items addressing *R*. All items were scored on a Likert scale from 1 to 4 (i.e. 1 = Strongly agree, 4 = Strongly disagree), and negatively formulated items were reversibly coded (i.e. high scores reflected high efforts as well as high rewards).

ERI was constructed in two ways. First, tertiles were calculated for *E* and *R* scores, respectively (low, intermediate and high), where the highest tertile of *E* and the lowest tertile of *R* were defined as high-risk ERI conditions. Second, the effort–reward ratio (*E*/*R* ratio) was calculated using sum scores of efforts (*E*) and rewards (*R*) and a correction factor (*c*) as follows [16]:


$$\begin{array}{l} E/R\;ratio\;=\;\left(E/R\right)\times c \end{array}$$

The correction factor (i.e. *c* = 7/3) adjusts for the difference in the number of items in each scale, creating a mean for *E* and *R*, respectively. An imbalance in experienced E versus R, rendering an *E*/*R* ratio > 1.00, was defined as a high-risk ERI condition as it implies an increased risk of burnout [10]. Accordingly, a dichotomized variable for exposure to a high-risk *E*/*R* ratio was created (i.e. 1 for *E*/*R* ratio > 1.00, 0 for *E*/*R* ratio ≤ 1.00). In the present study, the Cronbach’s alpha (*α*) values were 0.748, 0.784 and 0.796 for *E*, *R* and all 10 items of ERI, respectively.

In the present study, the risk of burnout was measured using the burnout assessment tool (BAT) constructed by Schaufeli *et al*. [17]. The BAT is a highly reliable and valid construct, psychometrically tested with a clinical cut-off value [18]. Accordingly, the BAT enabled analyses of burnout risk based on a compound score (i.e. across all dimensions of burnout) [19], and hence enabled dichotomization of the outcome variable.

The BAT comprises 23 items across four dimensions (i.e. symptoms) of burnout [17]: exhaustion (8 items), mental distance (5 items), emotional impairment (5 items) and cognitive impairment (5 items). All items were scored on a Likert scale from 1 to 5 (i.e. 1 = No, never, 5 = Yes, most of the time). A grand mean for all 23 items of BAT was created (Cronbach’s *α* = 0.943). A mean BAT score of ≥2.59 implies a high risk of burnout, in line with recommendations by Schaufeli *et al*. [19]. Accordingly, a dichotomized variable for risk of burnout was created (i.e. 0 for mean BAT score of <2.59 indicating no risk, and 1 for mean BAT score ≥2.59 indicating prevalent risk).

Sociodemographic data and occupational characteristics comprised sex, age, having a partner and/or children, employment type, hierarchical position, work site, clinical specialty, clinical work experience (years), number of working hours per week and shift work. Age was divided into quartiles (i.e. 27–38, 39–45, 46–57 and 58–69 years). The hierarchical positions were divided into physicians in training (i.e. junior, intern and resident physicians), specialist physicians and consultants. Employment type was divided into public sector and private sector. Clinical specialties were categorized in line with the definition by the Swedish National Board of Health and Welfare and divided into eight main categories comprising separate sub-specialties. To achieve a more even distribution, sub-specialties representing ≥1% of the total amount of physicians were separated from their main group and regarded as individual specialties.

Statistics Sweden conducted initial analyses of responders versus non-responders and provided calibration weights for the entire population of Swedish physicians as of 2018 (*N* = 36 604). This allowed for analysis based on the total population of Swedish physicians within a 1.9 percentage error margin. Statistical analyses were performed using STATA version 17 (StataCorp, College Station, TX), SPSS version 28 and R version 4.2.2.

Descriptive characteristics comprising ERI scores (i.e. scores for *E* and *R*, and *E*/*R* ratio) and prevalent risk of burnout (i.e. mean BAT score ≥2.59) were calculated as frequencies and means across all background variables (i.e. sociodemographic and occupational characteristics). Univariate analysis was performed where each background variable was included in a single-variable logistic regression model. Variables with a statistically significant association in the univariate analysis were included in a multivariate binary logistic regression analysis to identify the main factors associated with burnout.

Exploratory analysis was conducted to investigate the linear relationships between exposure and outcome variables. Since the association was non-linear, binary logistic regression models were used to examine the associations between exposure to ERI and risk of burnout (primary outcome). First, crude analysis was performed by including ERI (i.e. tertiles of *E* and *R* scores, and a dichotomized *E*/*R* ratio, respectively) into single-variable logistic regression models one by one. Second, the associations were adjusted for background variables that exhibited significance in the crude analysis (i.e. sex, age, clinical specialty, work experience, and rank). Finally, tertiles of *E* and *R* were forced into a multivariable model to assess the independence of the predictor variables. Results were presented as odds ratios (ORs), and all statistical tests were two sided, where a *P*-value of ≤0.05 was regarded as statistically significant.

## Results

The mean *E*/*R* ratio among all physicians was 1.19, and a majority (62%) had a high-risk *E*/*R* ratio (i.e. *E*/*R* ratio >1.00; Table 1). Across sociodemographic groups, female physicians had a higher mean *E*/*R* ratio (1.22) and, more commonly, a high-risk *E*/*R* ratio (65%) versus male physicians (1.16 and 58%, respectively). Furthermore, physicians in the upper age quartile had a lower mean *E*/*R* ratio, and less reported high-risk *E*/*R* ratio (1.11 and 50%, respectively) than younger physicians.

**Table 1.** Effort–reward imbalance and burnout assessment tool scores across sociodemographic and occupational characteristics in Swedish physicians

		Effort–reward imbalance					Burnout assessment tool
	*n* (%)	Effort score	Reward score	*E*/*R*	*E*/*R* ≥ 1.0	BAT score	BAT ≥ 2.59
		Mean (SD)	Mean (SD)	Mean (SD)	%	Mean (SD)	%
Sex							
Male	1027 (45)	8.9 (2.0)	19.5 (4.0)	1.16 (0.48)	58	1.82 (0.60)	12
Female	1266 (55)	9.4 (1.9)	19.2 (3.9)	1.22 (0.48)	65	2.00 (0.61)	17
Age quartiles							
27–37	626 (27)	9.2 (1.9)	19.0 (3.9)	1.21 (0.49)	64	2.00 (0.63)	16
38–45	564 (25)	9.4 (1.9)	19.5 (3.7)	1.18 (0.41)	64	1.94 (0.60)	15
46–57	571 (25)	9.5 (1.9)	19.0 (4.1)	1.26 (0.49)	68	1.97 (0.63)	18
58–69	532 (23)	8.7 (2.2)	19.8 (4.2)	1.11 (0.51)	50	1.75 (0.54)	8
Partner							
Yes	2070 (90)	9.2 (2.0)	19.4 (4.0)	1.19 (0.48)	62	1.91 (0.60)	14
No	223 (10)	9.1 (2.0)	18.8 (4.2)	1.22 (0.52)	61	2.00 (0.69)	19
Children							
Yes	1454 (63)	9.3 (1.9)	19.3 (3.9)	1.21 (0.46)	64	1.93 (0.60)	15
No	839 (37)	9.0 (2.1)	19.4 (4.1)	1.17 (0.52)	58	1.89 (0.63)	13
Employment							
Public sector	1878 (82)	9.3 (1.9)	19.2 (3.9)	1.21 (0.47)	63	1.93 (0.62)	15
Private sector	415 (18)	8.9 (2.2)	19.7 (4.1)	1.14 (0.50)	55	1.88 (0.57)	11
Rank							
Junior-, intern and resident physician	722 (32)	9.1 (1.9)	18.9 (4.0)	1.20 (0.50)	61	2.00 (0.63)	17
Specialist physician	920 (40)	9.4 (2.0)	19.4 (3.9)	1.21 (0.47)	64	1.92 (0.61)	15
Consultant	651 (28)	9.1 (2.1)	19.6 (4.0)	1.16 (0.47)	60	1.83 (0.58)	11
Work site							
Primary care facility	923 (40)	9.3 (1.9)	19.5 (3.9)	1.19 (0.46)	62	1.94 (0.60)	15
University hospital	547 (24)	9.4 (2.0)	19.1 (4.0)	1.22 (0.47)	64	1.90 (0.61)	14
Non-university hospital	660 (29)	9.3 (1.9)	19.2 (4.0)	1.21 (0.49)	64	1.90 (0.60)	13
Other^a^	163 (7)	7.9 (2.3)	19.4 (4.2)	1.05 (0.55)	43	1.91 (0.66)	15
Clinical specialty							
Paediatrics and neonatology	100 (4)	9.0 (2.2)	20.3 (3.5)	1.08 (0.38)	59	1.76 (0.55)	12
Radiology and functional specialties	93 (4)	8.9 (1.9)	19.4 (3.9)	1.15 (0.49)	57	1.88 (0.57)	15
Emergency medicine	38 (2)	10.4 (1.4)	18.6 (3.9)	1.40 (0.58)	82	2.13 (0.62)	24
Family medicine	809 (35)	9.3 (2.0)	19.5 (4.0)	1.18 (0.46)	62	1.93 (0.60)	14
Infectious disease medicine	28 (1)	9.5 (2.0)	19.8 (3.2)	1.17 (0.40)	61	2.04 (0.79)	25
Oncology	36 (2)	9.2 (2.1)	19.7 (4.0)	1.17 (0.45)	67	2.00 (0.60)	14
Other base specialties^b^	72 (3)	8.3 (2.2)	19.1 (3.8)	1.08 (0.44)	47	1.82 (0.58)	13
Geriatrics	36 (2)	9.1 (1.7)	18.4 (3.7)	1.22 (0.37)	69	2.21 (0.81)	28
Internal medicine	69 (3)	9.7 (1.6)	19.2 (4.0)	1.27 (0.54)	74	1.91 (0.60)	14
Cardiology	42 (2)	9.7 (1.6)	18.5 (4.0)	1.30 (0.41)	76	1.90 (0.56)	7
Other internal medicine specialties^c^	114 (5)	9.3 (2.0)	18.9 (4.1)	1.24 (0.49)	62	1.87 (0.59)	12
Anaesthesiology	122 (5)	9.4 (1.9)	19.5 (3.7)	1.19 (0.42)	63	1.82 (0.57)	7
General surgery	72 (3)	9.6 (1.8)	18.6 (4.5)	1.32 (0.53)	68	1.91 (0.65)	14
Obstetrics and gynaecology	87 (4)	9.5 (1.8)	20.0 (3.7)	1.17 (0.44)	59	1.70 (0.46)	5
Orthopaedics	92 (4)	8.6 (2.4)	19.0 (3.7)	1.13 (0.50)	58	1.84 (0.58)	10
Ophthalmology	29 (1)	8.9 (2.0)	19.6 (4.9)	1.19 (0.56)	55	1.81 (0.50)	10
Ear, nose and throat	44 (2)	8.6 (2.1)	20.8 (4.3)	1.06 (0.59)	41	1.78 (0.58)	7
Other surgical specialties^d^	55 (2)	9.0 (2.0)	17.8 (4.3)	1.30 (0.61)	60	1.86 (0.57)	16
Laboratory specialties^e^	41 (2)	9.7 (1.8)	19.7 (3.8)	1.21 (0.42)	63	2.03 (0.60)	20
Neurological specialties^f^	50 (2)	9.2 (2.3)	19.3 (4.3)	1.19 (0.46)	64	1.85 (0.56)	14
Psychiatry	95 (4)	9.1 (2.1)	19.0 (4.3)	1.22 (0.53)	60	2.17 (0.70)	24
No specialization/in training^g^	169 (7)	9.0 (2.0)	18.8 (3.9)	1.22 (0.51)	59	2.04 (0.67)	22
Clinical work experience (years)							
<5	224 (10)	8.8 (2.0)	19.1 (4.0)	1.16 (0.50)	56	2.0 (0.64)	17
5–10	559 (24)	9.3 (1.8)	19.1 (3.8)	1.22 (0.47)	64	1.97 (0.61)	15
10–15	485 (21)	9.6 (1.9)	19.1 (4.0)	1.25 (0.46)	68	2.0 (0.64)	18
>15	1025 (45)	9.1 (2.1)	19.6 (4.0)	1.16 (0.49)	58	1.83 (0.58)	12
Working hours (average/week)							
<30	127 (6)	8.0 (2.2)	20.2 (3.8)	0.98 (0.42)	43	1.84 (0.64)	13
30–40	702 (31)	8.8 (2.1)	19.8 (3.8)	1.10 (0.46)	54	1.89 (0.59)	14
41–50	1232 (53)	9.4 (1.9)	19.2 (4.0)	1.23 (0.48)	64	1.92 (0.60)	14
>50	232 (10)	10.1 (1.7)	18.1 (4.2)	1.40 (0.49)	82	2.02 (0.66)	17
Regular shift work							
Yes	1548 (68)	9.4 (1.9)	19.3 (4.0)	1.22 (0.48)	65	1.92 (0.61)	15
No	745 (33)	8.8 (2.1)	19.4 (4.0)	1.14 (0.48)	54	1.90 (0.60)	14
Total	2293	9.2 (2.0)	19.3 (4.0)	1.19 (0.48)	61	1.92 (0.61)	14

^a^Private healthcare clinics, outpatient clinics (outside hospital), occupational health care (outside hospital).

^b^Occupational medicine, dermatology, clinical genetics, rheumatology, forensic medicine.

^c^Allergology, endocrinology, haematology, pulmonology, gastroenterology, nephrology.

^d^Paediatric surgery, hand surgery, vascular surgery, plastic surgery, thoracic surgery, urology,.

^e^Clinical bacteriology and virology, pharmacology, immunology, chemistry, microbiology, pathology.

^f^Clinical neurophysiology, neurosurgery, neurology, rehabilitation medicine.

^g^Assistant doctors, intern physicians, licensed physicians pre-specialization.

Across occupational characteristics, large variations of ERI between different clinical specialties were found. Specifically, the highest mean *E*/*R* ratio and proportion with high-risk *E*/*R* ratio were observed within emergency medicine (1.40 and 82%, respectively), followed by general surgery (1.32, 68%), other surgical specialties (i.e. paediatric surgery, hand surgery, vascular surgery, plastic surgery, thoracic surgery, urology) (1.30, 60%), cardiology (1.30, 76%), and internal medicine (1.27, 74%) (Table 1). The corresponding lowest scores were found within ear, nose and throat (1.06, 41%), paediatrics and neonatology (1.08, 59%), other base specialties (i.e. occupational medicine, dermatology, clinical genetics, rheumatology, forensic medicine) (1.08, 47%) and orthopaedics (1.13, 58%). Generally, higher ERI scores were observed among more junior physicians, physicians with few years of clinical work experience, and those reporting long working hours (>41 hours/week) or regular shift work.

In total, 14.4% of Swedish physicians were at risk for burnout (i.e. mean BAT score ≥2.59) (Table 1). Across sociodemographic groups, female physicians were at higher risk of burnout (17%) than male physicians (12%). Furthermore, the risk of burnout was observed at similar frequencies across different age groups (15%–18%), except in the upper age quartile (8%).

Across occupational characteristics, the risk of burnout varied between clinical specialties. Specifically, the highest risk of burnout was observed within geriatrics (28%), followed by infectious disease medicine (25%), emergency medicine (24%) and psychiatry (24%). A lower risk of burnout was observed within obstetrics and gynaecology (5%), ear, nose and throat (7%), and anaesthesiology (7%). In general, the risk of burnout was higher among junior physicians, physicians with few years of clinical work experience and those reporting long working hours (>41 hours/week) or regular shift work.

Relevant predictor variables for the risk of burnout that exhibited statistical significance in crude analysis comprised sex, age, clinical specialty, work experience, and rank (Table 2). These variables were considered potential confounders and were included in the adjusted models.

**Table 2.** Odds ratios (crude) for risk of burnout across sociodemographic and occupational factors in Swedish physicians

	OR	95% CI	*P*
Sex			
Male	1.0	1.12–1.99	0.006
Female	1.49		
Age quartiles			
27–37	2.29	1.46–3.58	<0.001
38–45	1.97	1.24–3.12	0.004
46–57	2.33	1.48–3.66	<0.001
58–69	1.0		
Partner			
Yes	1.0		
No	1.20	0.78–1.84	0.40
Children			
Yes	1.08	0.81–1.46	0.59
No	1.0		
Employment			
Public sector	1.38	0.94–2.04	0.1
Private sector	1.0		
Rank			
Junior-, intern and resident physician	1.95	1.35–2.81	<0.001
Specialist physician	1.73	1.21–2.48	0.003
Consultant	1.0		
Work site			
Primary care facility	1.0		
University hospital	0.82	0.58–1.15	0.25
Non-university hospital	0.78	0.56–1.09	0.14
Other^a^	1.14	0.66–1.97	0.65
Clinical specialty			
Paediatrics and neonatology	1.0		
Radiology and functional specialties	1.41	0.55–3.59	0.47
Emergency medicine	2.92	0.99–8.61	0.05
Family medicine	1.57	0.76–3.27	0.22
Infectious disease medicine	2.62	0.84–8.18	0.1
Oncology	0.71	0.22–2.35	0.58
Other base specialties^b^	1.33	0.46–3.89	0.60
Geriatrics	2.98	0.97–9.15	0.06
Internal medicine	1.60	0.56–4.59	0.38
Cardiology	0.81	0.18–3.68	0.78
Other internal medicine specialties^c^	1.23	0.48–3.15	0.67
Anaesthesiology	0.75	0.27–2.10	0.59
General surgery	1.92	0.72–5.17	0.20
Obstetrics and gynaecology	0.42	0.12–1.48	0.18
Orthopaedics	1.26	0.45–3.54	0.67
Ophthalmology	0.90	0.22–3.75	0.88
Ear, nose and throat	0.48	0.12–1.90	0.29
Other surgical specialties^d^	2.0	0.72–5.52	0.18
Laboratory specialties^e^	2.54	0.85–7.57	0.09
Neurological specialties^f^	1.77	0.60–5.28	0.30
Psychiatry	3.08	1.28–7.43	0.01
No specialization/in training^g^	2.85	1.26–6.43	0.01
Clinical work experience (years)			
<5	2.05	1.30–3.26	0.002
5–10	1.45	1.01–2.08	0.044
10–15	1.80	1.25–2.59	0.002
>15	1.0		
Working hours (average/week)			
<30	1.0		
30–40	1.09	0.56–2.14	0.80
41–50	1.19	0.62–2.27	0.60
>50	1.54	0.74–3.20	0.25
Regular shift work			
Yes	1.17	0.87–1.57	0.31
No	1.0		

^a^Private healthcare clinics, outpatient clinics (outside hospital), occupational health care (outside hospital).

^b^Occupational medicine, dermatology, clinical genetics, rheumatology, forensic medicine.

^c^Allergology, endocrinology, haematology, pulmonology, gastroenterology, nephrology.

^d^Paediatric surgery, hand surgery, vascular surgery, plastic surgery, thoracic surgery, urology.

^e^Clinical bacteriology & virology, pharmacology, immunology, chemistry, microbiology, pathology.

^f^Clinical neurophysiology, neurosurgery, neurology, rehabilitation medicine.

^g^Assistant doctors, intern physicians, licensed physicians pre-specialization.

The crude results in Table 3 show that exposure to ERI was associated with a high risk of burnout among physicians (high *E*: OR = 8.95, low *R*: OR = 11.22, *E*/*R* ratio > 1.0: OR = 11.10).

**Table 3.** Associations between effort–reward imbalance and risk of burnout in Swedish physicians

	Crude	Model 1	Model 2
	Single OR (95% CI)	Multi OR^a^ (95% CI)	Multi OR^b^ (95% CI)
Effort score			
Low (<9)	1.0	1.0	1.0
Medium (9–10)	3.37 (2.06–5.50)^*^	3.57 (2.19–5.82)^*^	2.78 (1.69–4.57)^*^
High (>10)	8.95 (5.58–14.36)^*^	9.78 (6.06–15.79)^*^	6.34 (3.86–10.40)^*^
Reward score			
Low (<19)	11.22 (6.61–19.04)^*^	10.76 (6.36–18.20)^*^	7.36 (4.30–12.61)^*^
Medium (19–21)	2.93 (1.63–5.27)^*^	2.92 (1.62–5.27)^*^	2.50 (1.39–4.51)^*^
High (>21)	1.0	1.0	1.0
Effort–reward ratio			
*E*/*R* ≤ 1.0	1.0	1.0	
*E*/*R* > 1.0	11.10 (6.29–19.60)^*^	11.43 (6.52–20.03)^*^	

^a^Model 2 adjusted for age, sex, working experience (years), rank and specialty.

^b^Job Effort and Reward scores (tertiles) forced into a multivariable model and adjusted for age, sex, working experience (years), rank and specialty.

^*^Significant findings (*P* < 0.001).

In Model 1 (Table 3), relevant predictor variables that exhibited significance in crude analysis (i.e. sex, age, clinical specialty, work experience, and rank) were added to separate models for *E*, *R* and *E*/*R* > 1.0, respectively. In this model, associations between high efforts, high-risk *E*/*R* ratio and risk of burnout increased (high *E*: OR = 9.78, *E*/*R* ratio > 1.00: OR = 11.43), whereas the association between low rewards and risk of burnout decreased (low *R*: OR = 10.76) (Table 3).

In Model 2, scores for *E* and *R* were forced into a multivariable regression model and adjusted for the same predictor variables as in Model 2. The associations between high efforts, low rewards and risk of burnout decreased but remained significant (high *E*: OR = 6.34, low *R*: OR = 7.36), indicating sufficient independence of the ERI variables and their associations with burnout risk (Table 3).

## Discussion

The present study aimed to depict work-related stress by investigating the associations between ERI and the risk of burnout among Swedish physicians. We found that a majority of Swedish physicians had a high *E*/*R* ratio, strongly associated with an increased risk of burnout. In addition, we identified that Swedish physicians were exposed to higher ERI than the Swedish general working population (i.e. *E*/*R* ratio 1.06–1.10) [20]. Generally, we identified large variations in the prevalence of risk of burnout and ERI across sociodemographic and occupational factors, where differences across clinical specialties were substantial.

This study has some limitations that should be addressed. First, the nature of the present cross-sectional study design precludes any causal conclusions between exposure and outcome, and hence, longitudinal follow-ups are warranted. Second, data collection occurred during the third wave of the COVID-19 pandemic (i.e. February to May 2021), known to have increased work stress among physicians globally [21,22]. In the present study, this could have affected the distribution of work-stress exposure across clinical specialties, as some specialties were more exposed to the effects of the pandemic. Third, data were gathered using self-report questionnaires that may imply bias, for example, common method variance. Fourth, although the response rate was sufficient to achieve the required power, many of the invited participants declined the survey. In the case of prevalent underlying patterns, our results may have been affected (e.g. highly stressed participants could be less prone to submit questionnaires, or vice versa). Last, factors that may influence individual experiences of work stress (e.g. personality traits, resilience to stress, individual stress coping strategies) were not considered. Future studies could improve by addressing such factors that may impact the individual response to work-stress exposure [23]. A major strength of the present study was a large representative sample of Swedish physicians and the use of calibrating population weights, enabling analyses based on all Swedish physicians within a 1.9 percentage error margin. Additional strengths included the use of a validated psychosocial stress assessment model (i.e. ERI) and a robust tool to assess the risk of burnout (i.e. BAT). The BAT provides a compound measurement for burnout, in contrast to the widely used MBI that is restricted to different measurements of each symptom [17,24]. Although no psychometric testing of BAT has been performed in Sweden, it has been assessed in several other countries (e.g. Finland, Netherlands, Belgium, etc.) with one cut-off value [18]. Accordingly, in the present study, this cut-off value was applied.

Our findings align with previous studies reporting high levels of work-related stress among physicians [3,11,12,25]. For example, among Irish hospital-based physicians, a mean *E*/*R* ratio of 1.40 was identified [26], and 26% of Norwegian physicians had a high-risk *E*/*R* ratio (i.e. *E*/*R* > 1.00) [27]. In addition, strong associations between ERI and the risk of burnout among physicians have been reported previously [28]. However, we found no previous Swedish studies to compare data with. Specifically, the reward aspect of the ERI model had the strongest association with the risk of burnout, in line with previous findings [28]. A possible explanation for the strong link between low rewards and increased risk of burnout among physicians could be individual opportunities to influence efforts, as opposed to rewards. For example, during the early years of medical education, medical students are introduced to high efforts (e.g. a high share of mandatory educational elements, clinical rotations, etc.). In addition, for a relatively long period during their career, physicians are continuously exposed to high job efforts (e.g. shift work, long working hours, etc.). Hence, physicians may be more prone to accept high efforts as a natural and relatively stationary part of their job duties throughout their careers. In contrast, job rewards could be a more dynamic factor. This reasoning is strengthened by an acknowledged central role of rewards (i.e. economic rewards, esteem, status influence) in modern working life in general [29]. To achieve more detailed knowledge of ERI among Swedish physicians, further studies of personal experiences of work-related efforts and rewards are needed [13].

In previous studies, a high risk of burnout among physicians has been underlined [2,3], although comparisons of burnout risk prevalence between studies are extremely limited by methodological differences. For example, some studies have assessed only one dimension of burnout, that is, exhaustion [23,30], rendering large variations in burnout prevalence estimations (i.e. 0%–80.5%) [3]. The currently widespread methodological heterogeneity underlines the importance of a standardized application of burnout measurement tools [5,30]. Therefore, in the present study, we used BAT to measure burnout across all dimensions using established clinical cut-off values. This provided robust data that enable accurate comparisons in future follow-ups. Since burnout is a progressive condition [31], longitudinal studies of occupational health among physicians are needed to observe dynamic changes and ultimately implement preventive actions [2,31,32]. In conclusion, a majority of Swedish physicians were exposed to high levels of work-related stress, strongly associated with an increased risk of burnout. Generally, there was a high risk of burnout and high rates of ERI among Swedish physicians, with large variations across sociodemographic and occupational factors. Further studies are needed to explore variations of work-related stress and the risk of burnout across different clinical specialties. The present cross-sectional study underlines the need to monitor occupational health among physicians longitudinally.
